# Development and validation of parent-reported gastrointestinal health scale in MECP2 duplication syndrome

**DOI:** 10.1186/s13023-024-03022-2

**Published:** 2024-02-09

**Authors:** Davut Pehlivan, Sukru Aras, Daniel G. Glaze, Muharrem Ak, Bernhard Suter, Kathleen J. Motil

**Affiliations:** 1 Section of Pediatric Neurology and Developmental Neuroscience, Department of Pediatrics, Baylor College of Medicine, Houston, TX 77030 USA; 2 Blue Bird Circle Rett Center, Texas Children’s Hospital, Houston, TX 77030 USA; 3 Department of Genetics, Section of Metabolic Diseases, Children’s Hospital of Philadelphia, Philadelphia, PA 19146 USA; 4 Children’s Nutrition Research Center, Baylor College of Medicine, 1100 Bates Street, Houston, TX 77030 USA; 5 Department of Pediatrics, Baylor College of Medicine, Houston, TX 77030 USA

**Keywords:** *MECP2* duplication syndrome, Parent-reported outcome, Gastrointestinal health scale, Validity, Reliability

## Abstract

**Background/aims:**

We aimed to develop a validated patient-reported Gastrointestinal Health Scale (GHS) specific to *MECP2* Duplication Syndrome (MDS) to be used in clinical trials.

**Methods:**

MDS parents completed a Gastrointestinal Health Questionnaire (GHQ) to investigate the most relevant and important items associated with gastrointestinal problems in MECP2-related disorders. Item reduction was executed according to EORTC guidelines. We performed reliability and validity studies for the finalized scale.

**Results:**

A total of 106 surveys were eligible for item reduction and validation processes. The initial 55 items were reduced to 38 items based on parent responses, expert opinion, and initial confirmatory factor analysis (CFA). The final MDS-specific GHS included 38 items and 7 factors that underwent further reliability and validity assessments. The power of the study was at least 0.982. The Cronbach’s alphas of the instruments were General Health: 0.799, Eating-Chewing-Swallowing: 0.809, Reflux: 0.794, Motility: 0.762, Mood: 0.906, Medication: 0.595, Parenting: 0.942 and all items together: 0.928. The correlation coefficient between total and individual item scores ranged from 0.215 to 0.730. Because of the ordinal nature of the variables, the diagonal weighted least squares estimation (DWLS) method was used to execute the CFA and Structural Equation Modeling. The GHS had excellent model fit with the acceptable range of fit indices values.

**Conclusions:**

We developed a parent-reported, reliable, and valid MDS-specific GHS. This scale can be utilized in clinical settings or as an outcome measure in translational and clinical research.

**Supplementary Information:**

The online version contains supplementary material available at 10.1186/s13023-024-03022-2.

## Introduction

*MECP2* Duplication Syndrome, MDS (MIM# 300260), is a neurogenetic developmental disorder stemming from increased copies of the *MECP2* gene. The frequency of MDS has not been studied comprehensively. A recent study from Australia reported the prevalence as 0.65/100,000 live births [1]. The most common features include hypotonia, recurrent respiratory infections, developmental delay, epilepsy, and gastrointestinal and nutritional problems.

Currently, the management of MDS is symptomatic. However, preclinical studies using antisense oligonucleotide (ASO) provided robust phenotype recovery in mice models [2, 3]. Since disease-modifying treatments targeting the root problem are within reach, validated outcome measures for use in clinical and translational studies are needed. Toward this goal, we surveyed the caregivers of MDS individuals to explore the most bothersome complaints [4]. Gastrointestinal symptoms, especially constipation, were among the most bothersome problems and should be considered as primary outcome measures in future interventional studies.

Gastrointestinal problems are highly prevalent in MDS and allelic Rett syndrome (RTT, MIM 312750, caused by deletions or loss of function mutations of *MECP2*). To explore gastrointestinal health issues in MECP2-related disorders, we generated the Gastrointestinal Health Questionnaire. The reliability and validity studies for RTT were conducted and published separately [5].

In the current study, we aimed to develop a parent-oriented, reliable, and valid Gastrointestinal Health Scale (GHS) specific to MDS that could be utilized as an outcome measure in clinical assessments and interventional studies.

## Material and methods

### Gastrointestinal health questionnaire development and delivery

The study protocol was reviewed and approved by the Institutional Review Board (IRB) at Baylor College of Medicine with IRB approval number H-46176. We have created an online registry portal (https://mds.nrihub.org) that complies with the Health Insurance Portability and Accountability Act (HIPAA). This portal serves as a secure platform for conducting cross-sectional survey studies. All registrants were required to upload the genetic report confirming the molecular diagnosis of MDS. Our survey was promoted through the social media channels of family-based organizations. All participants provided written consent form for their registration to portal, participation into surveys and publishing the results.

Gastrointestinal problems are common in MECP2-related disorders including MDS and Rett. The senior author (K.J.M.) developed the Gastrointestinal Health Questionnaire (GHQ) through caregiver interviews and national surveys over the past two decades with multiple revisions based on feedbacks. The finalized GHQ was revised to make it comprehensive with no overlapping questions and understandable at the 8th-grade reading level. The GHQ consists of 55 questions on 9 factors, including General Health/Pain (5 questions), Eating/Chewing/Swallowing (9 questions), Reflux (3 questions), Gas/Bloating (5 questions), Diarrhea/Constipation (6 questions), Personality/Mood (5 questions), Medications (9 questions), Surgery (5 questions) and Parenting (8 questions). The responses were comprised of a five-point Likert scale from never to almost always except for the surgery questions where answers were “Yes/No”. Participants also were asked to report the relevance and importance of each question on a four-point Likert scale from not relevant/important to very relevant/important. The GHQ is a screening tool rather than a scale and investigates gastrointestinal problems broadly (e.g., both diarrhea and constipation questions were included in the GHQ). We applied GHQ to Rett syndrome and MDS patients and published overall gastrointestinal findings in these allelic disorders in separate articles [5, 6]. In this paper, we applied multiple statistical methods for the caregiver responses and removed irrelevant items. Now, this tool is called “MDS-Specific Gastrointestinal Health Scale” to be used as an outcome measure in clinical and translational research studies.

**Fig. 1:**
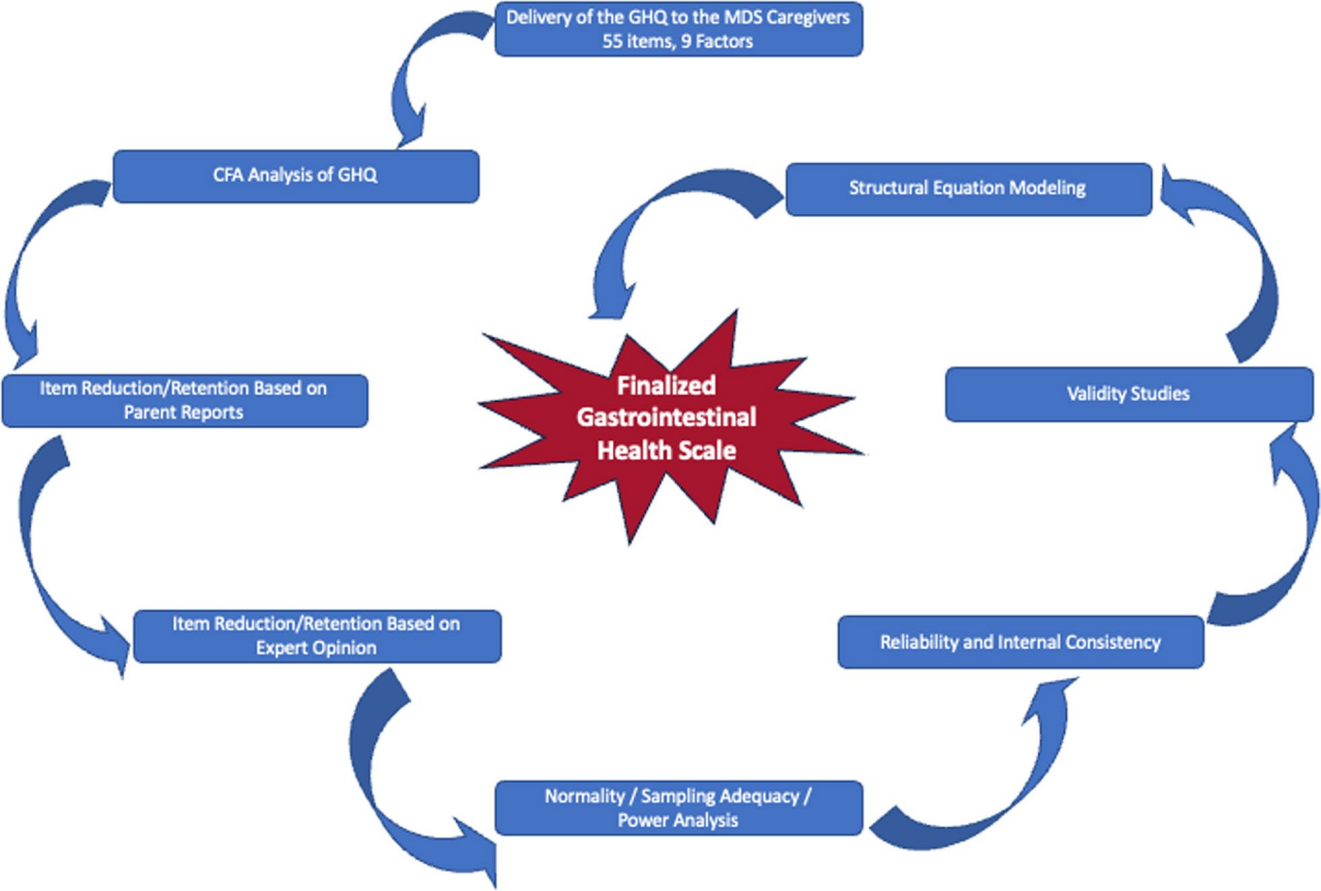
Flowchart of MDS Specific Gastrointestinal Health Scale Development Process. We initially surveyed MDS parents with GHQ. We then followed the described steps to create MDS-Specific GHS. *GHQ* Gastrointestinal Health Questionnaire, *GHS* Gastrointestinal Health Scale, *MDS* MECP2 Duplication Syndrome, *CFA* Confirmatory Factor Analysis

The survey was delivered to families between December 9th 2021 and January 20th 2022 through our secure portal. After the completion of the survey, we conducted statistical methods in two phases to tailor the GHQ specific to MDS (Fig. 1).

### Phase I: item reduction/retention

For Item Reduction, we performed a stepwise item elimination/retention process including a) Confirmatory Factor Analysis, b) parent-reported item elimination/selection and c) expert opinion.

#### Confirmatory factor analysis on the GHQ items

Confirmatory Factor Analysis (CFA) was executed on the initial GHQ items to examine the importance of items using factor loading values as a measure, then removing unrelated items from the questionnaire as the first step of item reduction. A factor loading score greater than 0.500 was determined as a cut-off according to Hu and Bentler’s guidelines [7]. We subsequently investigated whether the GHQ fits the CFA model by evaluating the following fit indices: Noncentrality-based Indices, Relative (Incremental) Fit Indices, and Absolute Fit Indices.

#### Item reduction/retention based on parent-reports

We used the fifth version of the guidelines developed by the European Organization for Research and Treatment of Cancer (EORTC) Quality of Life Group for a module development for the parent-based item reduction process [8]. We calculated floor effect, ceiling effect, compliance, relevance, importance, mean scores for relevance and importance, and prevalence ratio and prevalence scores for relevance and importance per guidelines.

The guideline recommended the following cut-off points for decision rules for selection of item reduction: Relevance: < 25% scored 1 (Although published guidelines stated score “0” instead of the score “1”, we have reached out to the authors of the guideline developers and confirmed that score should be “1”; they will provide a corrigendum to the Manual); Importance: > 60% scored3 or 4; Mean score > 1.5; Prevalence ratio > 30% or prevalence of scores 3 or 4 > 50%; Range > 2 points; No floor or ceiling effect: responses in categories 3&4 or 1&2 > 10%; and Compliance: at least 95% response to the item [8]. When we applied these criteria, we had too few items resulting in disruption of the structure of the survey. As suggested by the guideline, we modified Relevance as < 33% scored 1 and Importance as > 47% scored 3 or 4 [8]. This flexibility provided retention of additional items, thus regaining a model structure.

#### Item reduction/retention based on expert opinion

MDS experts (authors D.P., B.S., and K.J.M.) completed the item reduction process per EORTC guidelines. Experts were comprised of investigator clinicians who evaluate and manage MDS individuals at Texas Children’s Hospital Rett Center, a center of excellence dedicated to MECP2 and Rett-related disorders. MDS experts discussed the clinical importance of each removed and added items, regardless of factor loading scores and parent-based relevance and importance scores. A consensus was reached for the final scale for further statistical evaluations (phase II).

### Phase II: statistical evaluation of the MDS-specific gastrointestinal health scale for reliability and validity

#### Normality/sampling adequacy/power analysis

##### Normality

Prior to the validation and reliability analysis, normality was evaluated by the Kolmogorov–Smirnov test and Shapiro–Wilk test. The assumption of normality based on the skewness of values within the range ± 2 [9] and kurtosis of values within the range ± 7 [10] was determined. We also conducted Mardia’s Skewness Test and Mardia’s Kurtosis Test to investigate the multivariate normality of distribution.

##### Sampling adequacy

Kaiser–Meyer–Olkin (KMO) Test and Bartlett’s Test of Sphericity were used for data suitability and sampling adequacy The KMO test is a statistical measure to determine how data suit for factor analysis. The test measures sampling adequacy for each variable in the model and the entire model. Higher values mean a better fit of the data for factor analysis. KMO > 0.80 s was considered meritorious and less than 0.5 was unacceptable [11].

Bartlett’s test of Sphericity assesses the null hypothesis using an identity and correlation matrix. A significant statistical test (usually less than 0.05) shows that the correlation matrix is not an identity matrix (rejection of the null hypothesis). If the *p*-value from Bartlett’s Test of Sphericity is < 0.05, then our dataset is suitable for data reduction techniques such as principal component analysis and factor analysis studies.

##### Power analysis

We calculated power analysis according to RMSEA good fit indices criteria. We used three different methods for Post-hoc Power Analysis using “findRMSEApower”, “semPower.postHoc” and “semPower.compromise” functions in R language. Power of 0.80 and above is widely considered as a valid and acceptable value [12].

#### Reliability and internal consistency

Reliability and internal consistency, including factor-based internal consistency and overall internal consistency, of the scale was assessed by multiple methods including Cronbach’s alpha (value > 0.7 is considered meaningful) [13], McDonald’s Omega (value > 0.7 is considered meaningful) [13, 14], Consistent Reliability (RhoA) [15, 16], Composite Reliability (RhoC) [16, 17] and Spearman’s correlation analysis (*r* between 0.10–0.39 is considered a weak correlation) [18].

##### McDonald’s omega

McDonald’s omega is a reliability coefficient metric similar to Cronbach's Alpha [19]. McDonald’s Omega measures the strength of association between items and factors, and item-specific measurement errors. This provides more reasonable estimates compared to Cronbach's Alpha in reliability assessment [14]. The values and their interpretation are similar to Cronbach’s Alpha [19].

##### Composite reliability (RhoC)

Composite reliability (RhoC) is one of the primary reliability coefficients that uses the factor analysis method. Values between 0.60 and 0.90 are considered acceptable ranges and higher numbers indicate better reliability with the following ranges [17]:

- Values between 0.60 and 0.70: Acceptable,
- Values between 0.70 and 0.90: Satisfactory to good,
- Values above 0.90: Unacceptable. Because values above 0.9, especially above 0.95, indicate the presence of unnecessary items in the examined factor, thus disrupting the construct validity.

##### The reliability coefficient

The reliability coefficient (known as Exact Reliability or RhoA) is a relatively new method to assess the internal reliability of a scale. RhoA is usually a value between Cronbach’s alpha and composite reliability score. RhoA is an adjustment coefficient value to support the limitations of Cronbach’s alpha [15].

We further developed a new variable, a total item score, by summing all item scores. We calculated correlation coefficient values between this new variable and each factor’s item scores to assess the reliability.

#### Validity studies

##### Indicator collinearity

Indicator Collinearity was used to assess the correlation between factors and items of each factor. Variance Inflation Factor (VIF) is a standard measure to assess the collinearity. The VIF values of 5 or above indicate presence of collinearity problem. VIF values between 3 and 5 are acceptable but is not ideal The VIF values less than three suggest the absence of overlapping [16, 20].

##### Construct validity

We assessed the Construct Validity by calculating the Convergent Validity and Discriminant Validity.

##### Convergent validity

Convergent validity refers to the degree to which two measures of constructs that theoretically should be related, are in fact related [21]. In convergent validity, larger and statistically significant factor loadings mean better convergent validity. Loading values > 0.5 are acceptable values.

We further assessed convergent validity by Average Variance Extracted (AVE). If the AVE value is > 0.50, convergent validity is statistically established.

##### Discriminant validity

Discriminant validity tests whether concepts or measurements that are supposed to be unrelated are, in fact, unrelated [21]. It shows that constructs in the study have their own individual identity and are not too highly correlated with other constructs in the study. We assessed the discriminant validity of the GHS through the heterotrait-monotrait ratio (HTMT) of the correlations and Fornell and Larcker Criterion [22].

HTMT correlation assesses the arithmetic or geometric mean correlation among items across factors relative to the geometric-mean correlation among items within the same factor. The resulting HTMT values are interpreted as estimates of inter-construct correlations. Values more than 0.90 indicates the absence of discriminant validity, thus values less than 0.90 was considered as accepted [23].

Fornell and Larcker Criterion evaluates the factors in the model by calculating the square root of AVE in the diagonal with the correlation coefficients (off-diagonal) for each construct in the relevant rows and columns. This value should be greater than its correlation with all other factors.

##### CFA for finalized GHS

CFA is a multivariate statistical procedure that tests how well the measured items represent the number of factors. We performed CFA by using the Diagonally Weighted Least Squares (DWLS) method as an estimator to test and evaluate our model’s validity and whether the data fit a hypothesized measurement model. Based on the assumption of multivariate normality is severely violated and/or data are ordinal, the DWLS method provides more accurate parameter estimates [24–26]. We conducted CFA for the final MDS-specific GHS by calculating Fit Indices. We used the most common and well-known fit indices under four major categories to assess the construct of the model:

**Noncentrality-based indices**: RMSEA, CFI, RNI

1. The root mean square error of approximation (RMSEA) shows the lack of fit per degree of freedom of the model on the ground of sample size. Values < 0.05 indicate a very good fit. Of note, RMSEA is the only fit indices with a confidence interval value.
2. Comparative Fit Index (CFI) compares the sample covariance matrix with a null model. Accepted values > 0.90 mean a better fit.
3. Relative Noncentrality Index (RNI): Accepted values are same as CFI values.

**Relative Fit Indices**: IFI, TLI and NFI

1. Bollen’s Incremental Fit Index (IFI): Values > 0.90 indicates a better fit.
2. Tucker-Lewis Index (TLI) adjusts for the number of model parameters and values and the interpretation of the values are same as CFI.
3. Bentler-Bonett Normed Fit Index (NFI): Values and interpretation of the values are the same as CFI.

**Absolute Fit Indices:** Chi-square, GFI, AGFI, WRMR/SRMR

1. Chi-square and Chi square/df ratio (χ2/df): Chi-squared goodness-of-fit statistic measures the overall model fit to observed data; a significance test with *p*-values > 0.05 indicates a good fit. Χ2/df values of < 3.0 is considered acceptable.
2. Goodness of Fit Index (GFI): Evaluates the fitness between the proposed model and observed covariance matrix. Similar to IFI, a value > 0.95 is an acceptable value.
3. Adjusted Goodness of Fit Index (AGFI): Corrected GFI. Values > 0.90 are considered as an ideal value.
4. Weighted Root Mean Square Residual (WRMR)/Standardized Root Mean Square Residual SRMR: WRMR and SRMR measures the average differences between samples and population variances. However, SRMR is for continuous items and situations with large sample sizes. On the other hand, WRMR is for categorical items and preferred for relatively small sample sizes. Thus, in this study, we used the WRMR fit index instead of SRMR [27–29]. WRMR scores between 0.90 and 1.00 are considered appropriate values [30].

Finally, we measured the Efficiency Converges which calculates the number of iterations using R studio. Ideal Efficient Converge means reaching an optimum solution (efficient algorithm) after a few iterations. Thus, a lower number of iterations indicates a better model. Our iteration number is 7, which is proving the desired accuracy of our model.

### Phase II structural equation modeling (SEM)

We performed Structural Equation Modeling (SEM) using DWLS as an estimator to evaluate factors affecting parenting. For SEM, we evaluated the same fit indices with their reference values that we used in CFA to confirm whether our model fits.

### Phase II exploratory graph analyses (EGA)

EGA is a relatively new method to estimate the number of factors/dimensions and items with their relations to each other [31, 32]. We applied EGA to compare the final MDS-Specific GHS with the EGA’s proposed model.

All statistical analyses are conducted using multiple software and programs including SPSS version 29.0, JASP version 0.14.1.0 software (JASP Team, Amsterdam, Netherlands), JAMOVI version 2.3 and R Studio program.

## Results

### Subjects

A total of 122 caregivers initially participated in the survey. After review, 106 surveys met the eligibility criteria and were included in the analysis. Sixteen surveys were excluded due to either the MDS individual was female (as they do not exhibit the classic clinical features of MDS) or because parents did not provide the required genetic report for their child. However, amongst the 106 eligible surveys, three of them were females since they had translocations to an autosome thus represented as classic MDS phenotype (selective X inactivation favoring the duplicated X chromosome). Of the 106 eligible surveys, responders comprised of mothers (n = 88), fathers (n = 17), or mothers and fathers together (n = 1). Surgery questions were removed because: 1) the response was dichotomous, thereby incompatible with the model structure and 2) parental relevance and importance choices excluded these questions.

### Phase I: item reduction/retention studies based on CFA, parent-reports and expert opinion

We conducted CFA to assess the importance of items and exclude nonrelevant items based on factor loading score. This step removed 11 items and one factor (Additional file 1: Fig. S1 and Additional file 3: Table S1, column I). At the end of this step, 44 items and 8 factors remained. We examined the CFA models results with chi-square, χ2/df, TLI, GFI, RMSEA, and WRMR. All results were within the expected ranges described in the Methods section and validated the model’s structure (Data not shown).

**Table 1:** Mean, standard deviation, skewness, kurtosis, Shapiro–Wilk, McDonald ω, Cronbach's α, Spearmen Correlation analysis, and Confirmatory Factor Analysis of MECP2 Duplication Syndrome Specific Gastrointestinal Health Scale

Items	Mean	Standard deviation	Skewness	Kurtosis	Shapiro Wilk	McDonald ω (If item deleted)	Cronbach's α (If item deleted)	Spearman correlation coefficient	*p*-value for Spearman	CFA gactor loadings	95% CI		*p*-value
											Lower	Upper	
General HealthQ1	1.500	1.148	.423	-.453	< .001	0.921	0.925	0.665	< .001	0.923	0.877	0.970	< .001
General HealthQ2	1.425	1.179	.220	-.952	< .001	0.921	0.925	0.648	< .001	0.907	0.861	0.953	< .001
GeneralHealthQ3	1.160	1.212	.830	-.288	< .001	0.923	0.926	0.532	< .001	0.660	0.604	0.717	< .001
ECSQ1	1.292	1.493	.815	-.772	< .001	0.924	0.926	0.520	< .001	0.785	0.725	0.845	< .001
ECSQ2	2.443	1.421	-.299	-1.271	< .001	0.924	0.926	0.536	< .001	0.736	0.677	0.795	< .001
ECSQ3	2.038	1.603	-.020	-1.559	< .001	0.923	0.926	0.605	< .001	0.780	0.718	0.843	< .001
ECSQ4	2.594	1.419	-.551	-1.017	< .001	0.927	0.929	0.293	0.002	0.375	0.306	0.444	< .001
ECSQ5	1.943	1.440	.179	-1.261	< .001	0.923	0.925	0.607	< .001	0.842	0.786	0.899	< .001
ECSQ6	1.698	1.318	.297	-.934	< .001	0.923	0.926	0.542	< .001	0.800	0.744	0.857	< .001
ECSQ7	1.274	1.760	.791	-1.256	< .001	0.925	0.927	0.524	< .001	0.804	0.742	0.867	< .001
ECSQ8	2.085	1.651	-.112	-1.616	< .001	0.928	0.930	0.290	0.003	0.340	0.272	0.407	< .001
RefluxQ1	1.509	1.361	.326	-1.138	< .001	0.923	0.926	0.526	< .001	0.847	0.787	0.908	< .001
RefluxQ2	0.906	1.109	1.344	1.333	< .001	0.923	0.926	0.455	< .001	0.842	0.782	0.902	< .001
RefluxQ3	1.283	1.225	.739	-.223	< .001	0.921	0.925	0.613	< .001	0.929	0.855	1.003	< .001
MotilityQ1	2.736	1.174	-.766	-.002	< .001	0.923	0.926	0.556	< .001	0.943	0.869	1.017	< .001
MotilityQ2	2.113	1.174	-.044	-.632	< .001	0.925	0.927	0.379	< .001	0.654	0.592	0.717	< .001
MotilityQ3	2.406	1.067	-.396	-.097	< .001	0.924	0.927	0.439	< .001	0.660	0.593	0.728	< .001
MotilityQ4	2.849	1.440	-.843	-.726	< .001	0.924	0.927	0.420	< .001	0.675	0.597	0.752	< .001
MoodQ1	1.226	1.098	.593	-.286	< .001	0.923	0.926	0.517	< .001	0.824	0.776	0.873	< .001
MoodQ2	1.236	1.038	.708	.180	< .001	0.922	0.925	0.629	< .001	0.926	0.881	0.972	< .001
MoodQ3	1.179	1.128	.734	-.264	< .001	0.923	0.926	0.556	< .001	0.863	0.819	0.906	< .001
MoodQ4	1.208	1.084	.626	-.171	< .001	0.923	0.926	0.464	< .001	0.838	0.792	0.884	< .001
MoodQ5	1.575	1.086	.416	-.199	< .001	0.923	0.926	0.518	< .001	0.860	0.815	0.906	< .001
MedicationQ1	0.670	1.144	1.769	2.199	< .001	0.925	0.927	0.466	< .001	0.650	0.564	0.735	< .001
MedicationQ2	1.189	1.768	.909	-1.115	< .001	0.925	0.927	0.524	< .001	0.858	0.762	0.954	< .001
MedicationQ3	0.755	1.459	1.656	.987	< .001	0.927	0.929	0.274	0.004	0.512	0.422	0.603	< .001
MedicationQ4	0.142	0.608	5.351	30.600	< .001	0.925	0.928	0.246	0.011	0.746	0.641	0.852	< .001
MedicationQ5	0.245	0.803	3.694	13.548	< .001	0.925	0.928	0.215	0.027	0.661	0.569	0.754	< .001
MedicationQ6	1.406	1.689	.583	-1.431	< .001	0.927	0.930	0.320	< .001	0.454*	0.371	0.537	< .001
MedicationQ7	2.302	1.730	-.288	-1.669	< .001	0.928	0.930	0.314	0.001	0.402*	0.320	0.484	< .001
ParentingQ1	2.038	1.129	.087	-.339	< .001	0.923	0.924	0.718	< .001	0.890	0.855	0.885	< .001
ParentingQ2	2.689	1.190	-.685	-.336	< .001	0.923	0.925	0.568	< .001	0.849	0.813	0.957	< .001
ParentingQ3	1.849	1.293	.179	-.974	< .001	0.923	0.924	0.709	< .001	0.926	0.895	0.833	< .001
ParentingQ4	2.726	1.276	-.646	-.633	< .001	0.923	0.925	0.592	< .001	0.791	0.750	0.925	< .001
ParentingQ5	1.858	1.558	.163	-1.509	< .001	0.923	0.925	0.639	< .001	0.893	0.861	0.953	< .001
ParentingQ6	1.575	1.387	.540	-.832	< .001	0.923	0.924	0.694	< .001	0.921	0.890	0.922	< .001
ParentingQ7	1.811	1.468	.242	-1.287	< .001	0.922	0.924	0.730	< .001	0.888	0.854	0.928	< .001
ParentingQ8	2.472	1.402	-.448	-.997	< .001	0.923	0.925	0.653	< .001	0.893	0.859	0.925	< .001

CFA: Confirmatory Factor Analysis; CI: Confidence Interval; ECS: Eating-Chewing-Swallowing; Q: Question

^*^Factor loadings below 0.5 are bolded

We applied the EORTC recommended relevance (score 1 < 25%) and importance (score 3 or 4 > 60%) cut-offs for the entire GHQ (Additional file 3: Table S1). Thirty-nine out of 55 questions were eliminated with these criteria (Additional file 1: Fig. S1 and Additional file 3: Table S1, columns B and C). The remaining 16 items were too few and disrupted the survey structure. We used the flexibility option in the guidelines and relaxed the relevance criteria from < 25% to < 33% for score 1 and the importance criteria from > 60% to > 47% for scores 3 and 4 without changing other criteria (Mean, Prevalence ratio, Range and Floor effect or Ceiling effect). The relaxed criteria restored an additional 14 questions to achieve a total of 30 questions (Additional file 1: Fig. S1 and Additional file 3: Table S1, columns D and E).

The experts gathered to discuss each item reduction result, regardless of parent-based responses and CFA results. The final GHS, which included a total of 38 items with 7 factors. This scale is called the MDS-specific Gastrointestinal Health Scale (GHS) and underwent reliability and validity testing (Additional file 1: Fig. S1 and Additional file 3: Table S1, column K).

### Phase II: reliability and validity studies

#### Normality, sampling adequacy and power analysis

Kolmogorov–Smirnov and Shapiro–Wilk tests revealed that the data distribution was not normal. When considering the skewness normal range between -2 and + 2 and kurtosis normal range between -7 and + 7, skewness and kurtosis values for all items were within expected ranges except Questions 4 and 5 in the Medication factor for both skewness and kurtosis values (Table 1).

Multivariate normality analysis using Mardia’s Skewness Test and Mardia’s Kurtosis Test showed Skewness and Kurtosis values for Mardia’s Coefficients, Kappa and *p*-values are 633.825 and 1542.931 for Mardia’s Coefficient, 11,197.575 and 2.141 for Kappa, and < 0.001 and 0.032 for *p*-values, respectively.

Sampling adequacy measurements were assessed with KMO [KMO value** = **0.834 which is above Kaiser’s (703) = 2553 (*p*-value < 0.001)]. This result indicates strong sampling adequacy for the CFA. Bartlett’s Test of Sphericity analysis resulted in a Chi-square of 2553.034 (*p*-value < 0.001), which showed that our scale is suitable to execute factor analyses.

We calculated the Power of the gastrointestinal health scale using the CFA model-derived degree of freedom and sample size, and RMSEA good fit values. Power calculation using Basic Power Analysis, Post-hoc Power Analysis, Compromise Power Analysis revealed 0.999, 0.994 and 0.982, respectively, confirming the strong power of the study.

#### Reliability and internal consistency

Factor-Based Internal Consistency: We calculated Cronbach’s alpha, McDonald’s omega, RhoA and RhoC values for each factor to assess the reliability. All factor reliability values were over 0.700 except Medication Factor, which confirms that each factor’s internal consistency was very good except for Medication (Table 2 and Fig. 1).

**Table 2:** Factor based reliability and AVE of gastrointestinal health scale

Factors	Cronbach’s alpha	McDonald’s omega	RhoA	RhoC	AVE
General health	0.801	0.816	0.833	0.884	0.720
ECS	0.814	0.817	0.857	0.859	0.444
Reflux	0.800	0.810	0.804	0.882	0.713
Motility	0.768	0.768	0.788	0.852	0.591
Mood	0.906	0.906	0.913	0.930	0.727
Medication	0.638	0.602	0.658	0.761	0.318
Parenting	0.943	0.944	0.945	0.953	0.716

*AVE* Average variance extracted; *RhoA*: Reliability coefficient; *RhoC* Composite reliability; *ECS* Eating-chewing-swallowing

We calculated the Composite reliability (RhoC) values as a composite reliability measure. RhoC values were between satisfactory to good except for two factors (mood and parenting) with values between 0.90 to 0.95.

The Reliability Coefficient (Exact Reliability or RhoA) value for the factors in our scale had values above 0.70 except for medications (0.658), however, RhoA and RhoC values were higher than Cronbach’s alpha.

Overall Internal Consistency: To assess the Overall Internal Consistency, we calculated Cronbach’s alpha and McDonald’s omega values for all factor items together. Cronbach’s alpha and McDonald’s omega were 0.928 (95% confidence interval 0.907–0.946) and 0.926 (95% confidence interval 0.905–0.946), respectively, which means excellent coefficient scores.

Spearman’s Correlation Analysis: We examined the correlation between each item and the total item score (Sum of items) using Spearman’s correlation. All pairwise correlation coefficients were statistically significant [*p*-values mostly < 0.001 with the highest *p*-value of 0.027, see Table 1 for entire item values].

#### Validity studies

##### Indicator collinearity

All VIF values were under 5. VIF values were also under 3 in 6 out of 7 factors except for some of the parenting items (Additional file 4: Table S2).

##### Construct validity

Convergent validity assessment as part of the construct validity is conducted by calculating factor loading (Table 1) for each item and AVE values for each factor (Table 2). Factor loading values were mostly very high except for four items between 0.34 and 0.50, which were retained in the scale by the expert opinion (Table 1). AVE values for the factors in our scale had values above 0.50 except for eating-chewing-swallowing function (0.444) and medications (0.318).

Discriminant Validity: We calculated HTMT, and Fornell and Larcker Criterion scores to assess discriminant validity. All HTMT values were within the acceptable range and less than 0.90, confirming the discriminant validity of the scale (Table 3).

**Table 3:** HTMT: heterotrait–monotrait (ratio of correlations method)

Factors	General health	ECS	Reflux	Motility	Mood	Medication	Parenting
General health	0.000	0.000	0.000	0.000	0.000	0.000	0.000
ECS	0.532	0.000	0.000	0.000	0.000	0.000	0.000
Reflux	0.699	0.586	0.000	0.000	0.000	0.000	0.000
Motility	0.559	0.394	0.455	0.000	0.000	0.000	0.000
Mood	0.723	0.451	0.529	0.456	0.000	0.000	0.000
Medication	0.562	0.628	0.637	0.422	0.333	0.000	0.000
Parenting	0.687	0.445	0.551	0.597	0.473	0.514	0.000

All Fornell and Larcker values were within Fornell and Larcker Criterion for each factor, further supporting the discriminant validity of our scale (Table 4).

**Table 4:** Fornell and Larcker criterion

Factors	General health	ECS	Reflux	Motility	Mood	Medication	Parenting
General Health	0.848*	0.000	0.000	0.000	0.000	0.000	0.000
ECS	0.460	0.666*	0.000	0.000	0.000	0.000	0.000
Reflux	0.572	0.507	0.845*	0.000	0.000	0.000	0.000
Motility	0.458	0.312	0.359	0.769*	0.000	0.000	0.000
Mood	0.620	0.409	0.468	0.395	0.852*	0.000	0.000
Medication	0.421	0.441	0.479	0.268	0.251	0.564*	0.000
Parenting	0.602	0.419	0.488	0.518	0.444	0.411	0.846*

*ECS* Eating-Chewing-Swallowing

*This value should be greater than its correlation with all other factors

##### Confirmatory factor analysis

**Fig. 2:**
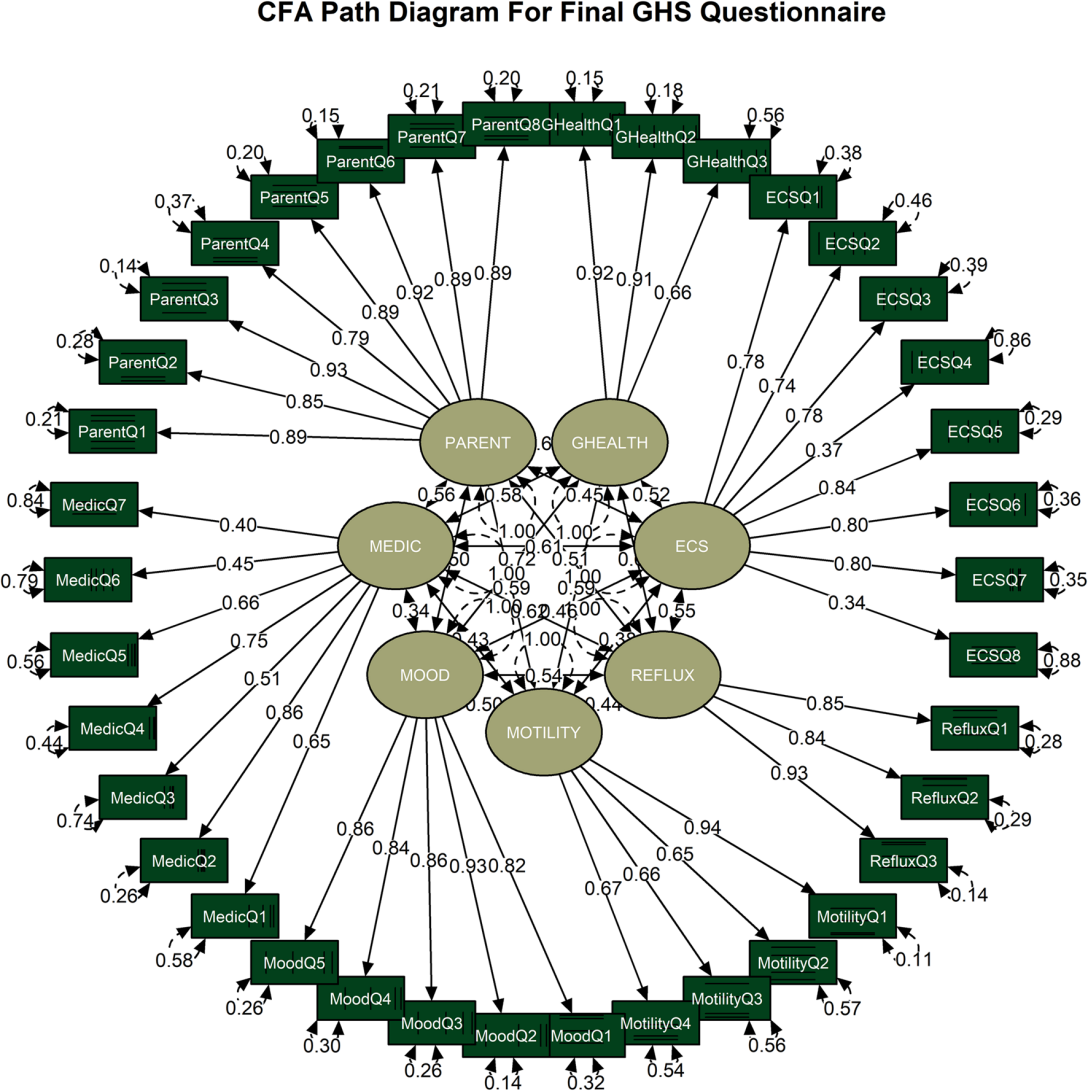
Path Diagram for the GHS. Items are shown in rectangles and Factors are shown in oval shapes. Factor loading values are shown on the arrows from Factors to Items. Item Residual values are given with the numbers next to items. Factor correlation values are provided with the arrows between Factors. *ECS* Eating-Chewing-Swallowing, *GHealth* General Health, *Medic* Medication, *Q* Question, *Parent* Parenting

The CFA of the final MDS-specific GHS showed a perfect model fit based on the goodness of fit statistics. Chi-square was 708.251 with a df value of 644 (n = 106) and the *p*-value was 0.04. The χ2/df fit value as 1.099 (acceptable value < 3). We calculated 10 different fit indices, and eight out of nine indices were within the acceptable values including the most commonly used ones: CFI 0.997 (acceptable value > 0.85), RMSEA 0.031 [Confidence Interval 90%: 0.007 – 0.044], GFI 0.975 (acceptable value > 0.85). The only fit index that was not within the acceptable value was SRMR 0.097 (preferred value < 0.08). All fit indices scores and their acceptable values were detailed in Table 5. Path diagram CFA is shown in Fig. 2.

**Table 5:** Fit Indices of MECP2 Duplication Syndrome Specific Gastrointestinal Health Scale

Fit indices*	Χ2	Χ2/df	RMSEA	CFI	RNI	IFI	TLI	NFI	GFI	AGFI	WRMR
Value	*p*-value = 0.04	1.099	0.031 CI 90% (0.007–0.044)	0.997	0.997	0.997	0.997	0.971	0.975	0.967	0.911
Acceptable value	*p*-value > 0.05	< 3	Good Fit ≤ 0.05	> 0.90	> 0.90	> 0.90	> 0.90	> 0.90	> 0.95	> 0.90	0.90–1.00

*Fit Indices

Χ^2^: Chi -Square, Χ^2^/df: Chi-Square Degrees of Freedom divided, RMSEA: Root mean square error of approximation, CFI: Comparative Fit Index, RNI: Relative Noncentrality Index, IFI: Bollen’s Incremental Fit Index, TLI: Tucker-Lewis Index, NFI: Bentler-Bonett Normed Fit Index, GFI: Goodness of Fit Index, AGFI: Adjusted Goodness of Fit Index, WRMR: Weighted Root Mean Square Residual

#### Structural equation modeling

SEM analysis revealed three factors independently affect parenting including general health, motility and medications with *p*-values < 0.001, < 0.001 and 0.04, respectively.

#### Exploratory graph analysis (EGA)

We explored whether our model (CFA-based MDS-specific GHS) overlaps with the proposed EGA model. The EGA identified six factors with 37 items. Importantly, 34 out of 37 questions were present in our GHS (~ 92% overlap with the existing scale), supporting our model structure and providing further evidence that EGA should be considered as an adjunct or alternative method for exploratory factor analysis.

## Discussion

In this study, we developed an MDS-specific gastrointestinal health scale (MDS-specific GHS) based on CFA, parents’ responses and experts’ opinions. The final scale included 38 items in 7 factors and covers most bothersome gastrointestinal symptoms. The statistical studies revealed that the MDS-specific GHS is a reliable and valid tool developed based on parent-reports. Thus, this survey can be used as an outcome measure of symptom severity in clinical and translational research studies. Moreover, since it is easy and quick to apply, it can serve as a screening tool for individuals with MDS in gastrointestinal clinics.

Outcome measures are tools to assess the patient’s severity of symptoms in an objective way. Outcome measures are more valuable if patients or caregivers are involved in the development process of tool development [33]. MDS individuals are not the source of information in our surveys due to their limited or absent communication skills stemming from their profound cognitive deficits. Thus, parents/caregivers were the primary source of information.

We followed a stepwise method in our scale development. First, we conducted item-reduction on the entire GHQ using CFA, EORTC guideline decision rules and expert opinion. The CFA model removed 12 items and one factor. Applying the EORTC decision rules disrupted the survey structure. Thus, we loosened the relevance and importance criteria per the EORTC guideline [8], resulting in 31 items. Finally, experts included additional 7 items, resulting in a total of 38 items and 7 factors for the final GHS.

The power of our study was measured by three functions using R-language. The lowest score amongst them was 0.982 (Compromised Power Analysis) proving the power of the study. Furthermore, sampling adequacy assessment (KMO and Bartlett’s Test of Sphericity) showed the suitability of the scale for factor analysis. Skewness/kurtosis values were low for two items in the Medication factor. However, these two items were included in the final scale per expert opinion.

**Fig. 3:**
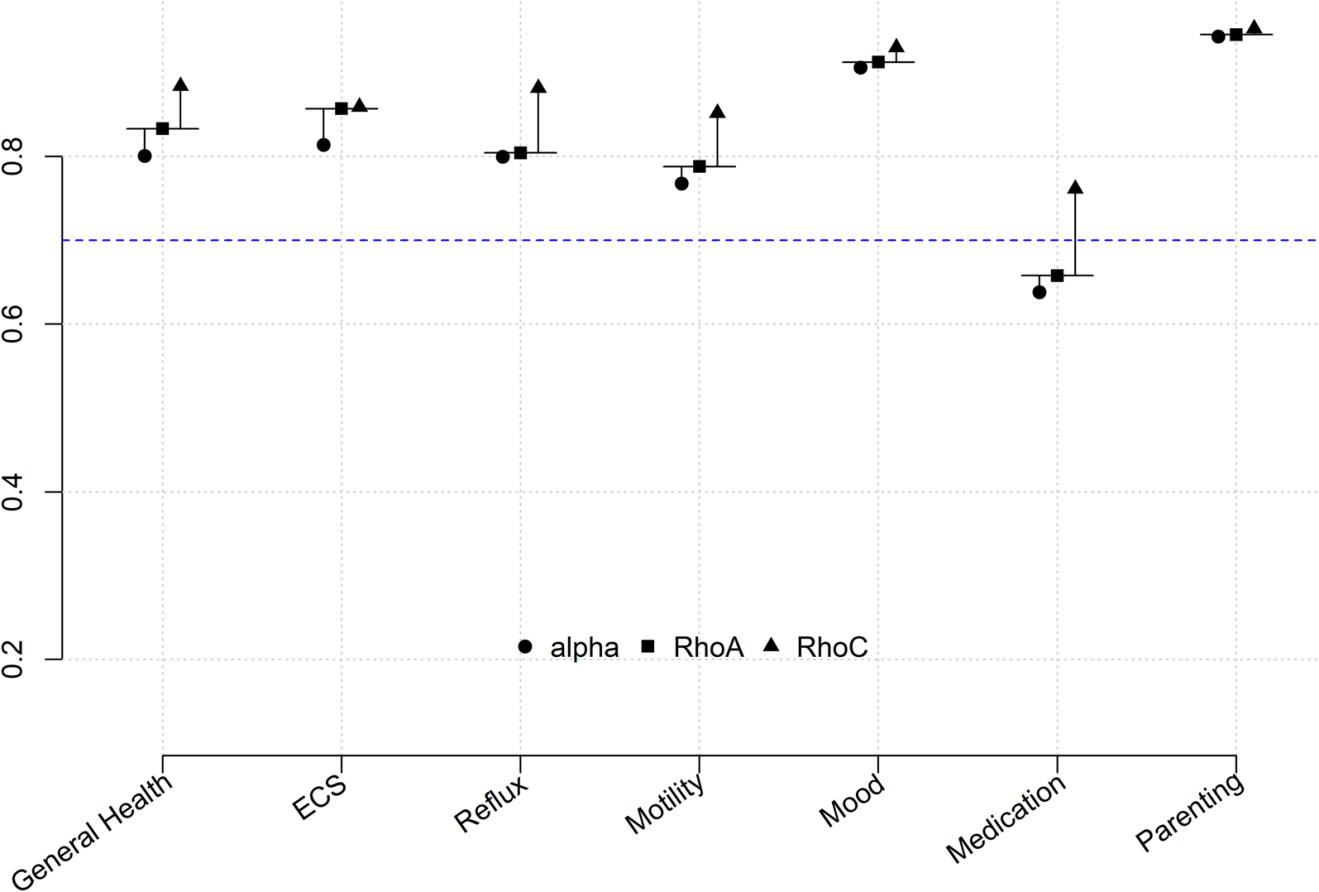
Reliability Assessments of the GHS. Cronbach alpha, RhoA and RhoC values for each factor. All values are within desired values except for Medication factor where Cronbach alpha and RhoA are below perfect value

The reliability of our study is assessed by multiple measures including Cronbach’s alpha, McDonald’s omega, composite reliability (RhoC) and exact reliability (RhoA) as opposed to many other studies which mostly conduct reliability analysis based on Cronbach’s alpha. All these reliability measures have limitations thus measuring reliability with multiple methods provided a more robust reliability assessment for our model. One of the important but underestimated constraints of Cronbach’s alpha is that it assumes all items’ loadings are the same in the population, thus providing lower reliability values [16]. On the other hand, very high (> 0.95) RhoC values can provide information on construct validity. Thus, Cronbach’s alpha assesses the lower bound whereas RhoC assesses the upper bound for internal consistency reliability [16] In our scale, the Medication factor’s Cronbach Alpha and RhoA scores were borderline low, 0.638 and 0.658, respectively (Fig. 3). This is likely due to lower skewness scores for two items in the medication factor and experts retained them in the survey due to clinical importance. Additionally, even if these two medication items were removed from the scale, overall Cronbach’s alpha changes were minimal (Table 1).

We performed validation studies with construct validity, discriminant validity and CFA. In CFA, the *p*-value for the Chi-square was 0.04. However, the *p*-value should not be statistically significant (> 0.05), which is an indicator of good model fit. This was a commonly encountered problem in CFAs, thus fit indices values were developed [34]. We calculated 10 different fit indices and all of them were within acceptable values. We used the estimator DWLS when we were conducting CFA analysis. In DWLS, WRMR is more meaningful than SRMR and our WRMR value is also within acceptable values. We thus removed SRMR from our fit indices list. Eventually, all of our fit indices including the most important and commonly used ones (χ2/df, RMSEA, CFI, TLI, GFI and WRMR) were within acceptable ranges (Table 5).

Factor loading scores and AVE values were used to evaluate construct validity. Both analyses showed borderline low values in the eating/chewing/swallowing and medication factors. There are two questions in each section (questions 4 and 8 in the eating/chewing/swallowing factor and questions 6 and 7 in the Medication factor) that has low factor loading and AVE values. Lastly, another key element of validity assessment is Discriminant Validity. Fornell–Larcker criterion has been in use as the primary criterion to assess discriminant validity. However, the HTMT criterion is becoming the preferred choice in recent years [16]. In our study, we calculated both HTMT, and Fornell and Larcker Criterion and both analyses were within expected ranges for discriminant validity. Overall, these analyses confirm the validity of our scale. Further evidence for the validity of our scale comes from EGA. Final MDS-Specific GHS (Additional file 2: Fig. S2A) and EGA’s proposed model (Additional file 2: Fig. S2B) were very similar (7 factors with 38 items versus 6 factors with 37 items) despite multiple items being reincorporated into the actual scale with expert opinion.

SEM analysis to identify factors affecting parenting revealed general health, motility and medications. Our meaningfulness survey also identified motility (constipation) as one of the top concerns that caregivers were seeking treatment for, which confirms the SEM analysis [4].

This study had limitations based on study design. The study was conducted as an online survey, rather than an in-person interview process, which could lead to bias. The study design was cross-sectional, rather than longitudinal, which also limits the exploration of the full scope of the symptoms and their severity. This design could have caused parental bias in their relevance and importance decisions. Furthermore, validation studies of the survey ideally should be conducted longitudinally. Most of our sample population originated from USA and Europe. This selection could cause bias in responses due to treatment preferences and the socioeconomic status of these countries. Finally, the present study was conducted during the COVID pandemic, which may have affected parental responses.

In conclusion, MDS-Specific GHS is a valid and reliable rating scale with adequate psychometric properties to measure the gastrointestinal health of MDS individuals. The significance of this scale lies in its development based-on parent-reports. It is reliable and valid tool, that is also easy to administer. This scale can serve as a valuable outcome measure in clinical trials and translational studies. Additionally, it can be utilized as a screening tool for gastrointestinal health in clinical settings.

### Supplementary Information

**Additional file 1: Figure S1**. Item Reduction Process of Gastrointestinal Health Questionnaire According to EORTC Guideline. GHQ: Gastrointestinal Health Questionnaire, MDS: MECP2 Duplication Syndrome, CFA: Confirmatory Factor Analysis, EORTC: European Organisation for Research and Treatment of Cancer.

**Additional file 2: Figure S2**. Exploratory Graph Analysis of the GHQ. A. Final GHS model based on Confirmatory Factor Analysis. **B.** Proposed Exploratory Graph Analysis model. ECS: Eating-Chewing-Swallowing, GHealth: General Health, Medic: Medication, Q: Question, Parent: Parenting.

**Additional file 3**. Details of the item reduction/retention studies based on CFA, parent-reports and expert opinion.

**Additional file 4**. Collinearity values for factors and each item of Gastrointestinal Health Scale.

## Data Availability

The results from the current study are available from the corresponding and/or first author on reasonable request.

## Abbreviations

AGFI: Adjusted goodness of fit index
AVE: Average variance extracted
CFA: Confirmatory factor analysis
CFI: Comparative fit index
DWLS: Diagonal weighted least squares
EORTC: European Organization for Research and Treatment of Cancer guidelines
EGA: Exploratory graph analyses
GFI: Goodness of fit index
GHQ: Gastrointestinal Health Questionnaire
GHS: Gastrointestinal health scale
HTMT: Heterotrait–monotrait ratio
IFI: Bollen’s incremental fit index
MDS: *MECP2* Duplication syndrome
NFI: Bentler–Bonett normed fit index
SEM: Structural equation modeling
RMSEA: Root mean square error of approximation
RNI: Relative noncentrality index
TLI: Tucker–Lewis index
WRMR: Weighted root mean square residual
Χ^2^: Chi-square
Χ^2^/df: Chi-square degrees of freedom divided

## Declarations

### Consents for publication

Surveyors provided consent for participation into the study and publication of results.
