# Supplementary figures and images for: Development and validation of parent-reported gastrointestinal health scale in MECP2 duplication syndrome
Source: Orphanet J Rare Dis. 2024 Feb 9;19:52. doi: 10.1186/s13023-024-03022-2 (PMC10854118; doi:10.1186/s13023-024-03022-2)

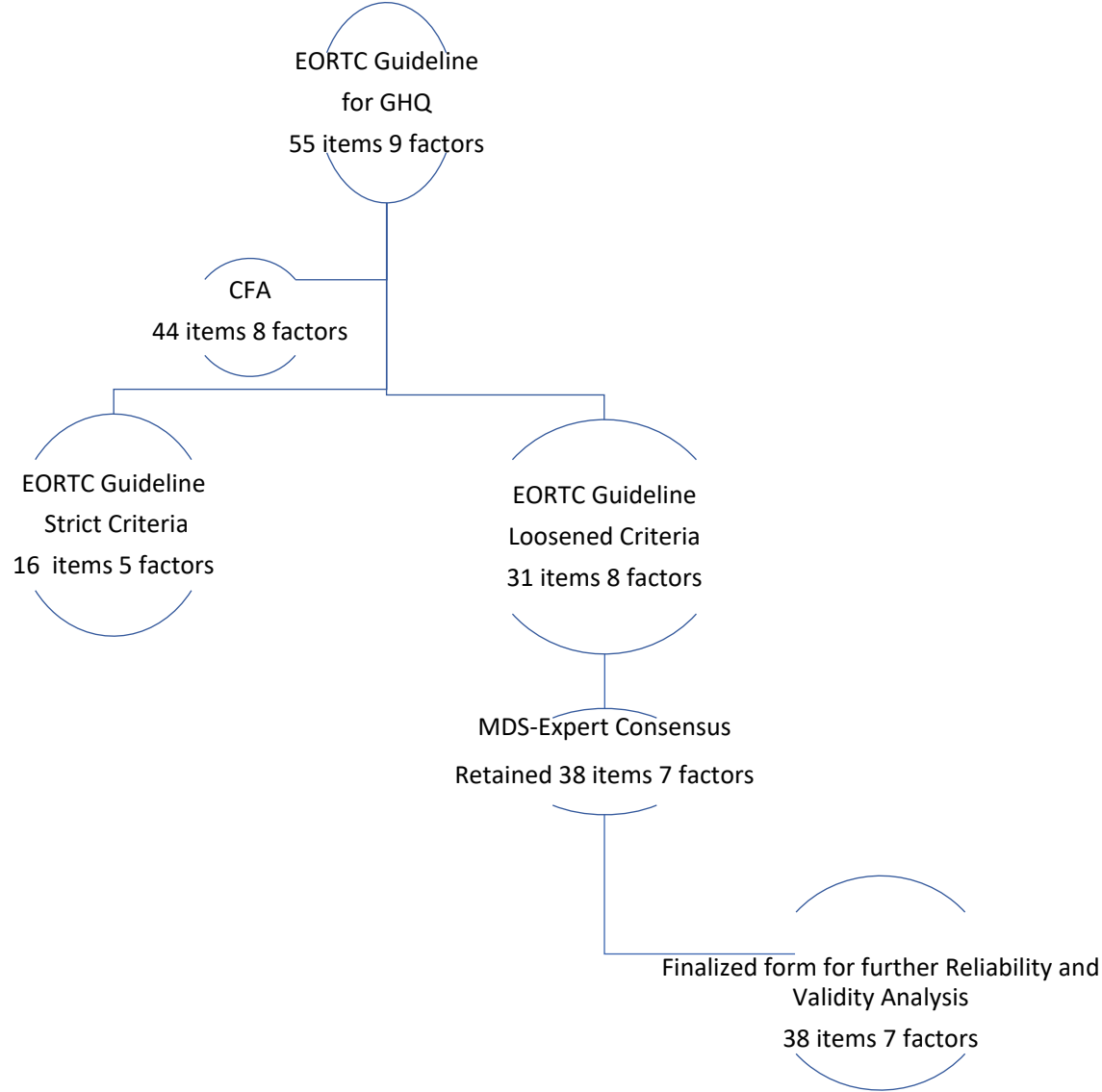

Supplement: Supplementary file 1 — Additional file 1: Figure S1. Item Reduction Process of Gastrointestinal Health Questionnaire According to EORTC Guideline. GHQ: Gastrointestinal Health Questionnaire, MDS: MECP2 Duplication Syndrome, CFA: Confirmatory Factor Analysis, EORTC: European Organisation for Research and Treatment of Cancer. [file 13023_2024_3022_MOESM1_ESM.pdf]

A)

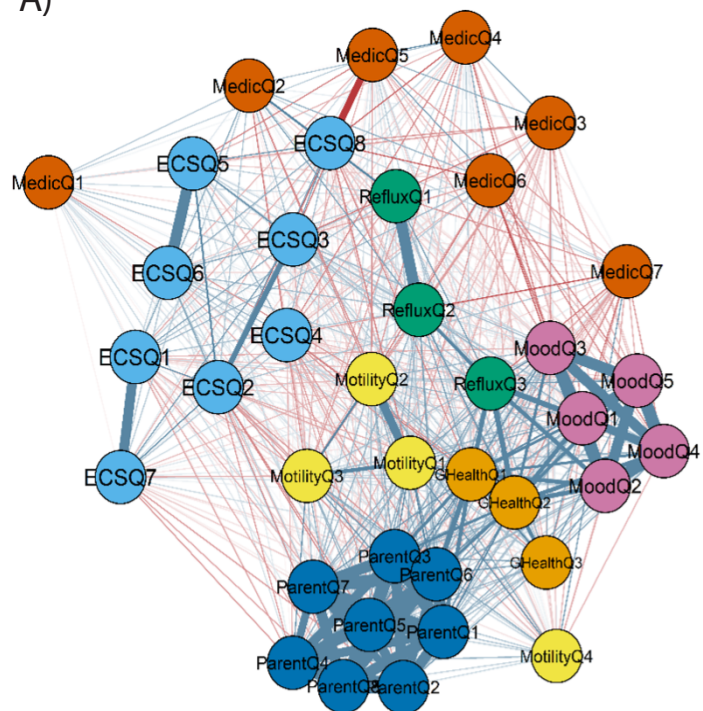

B)

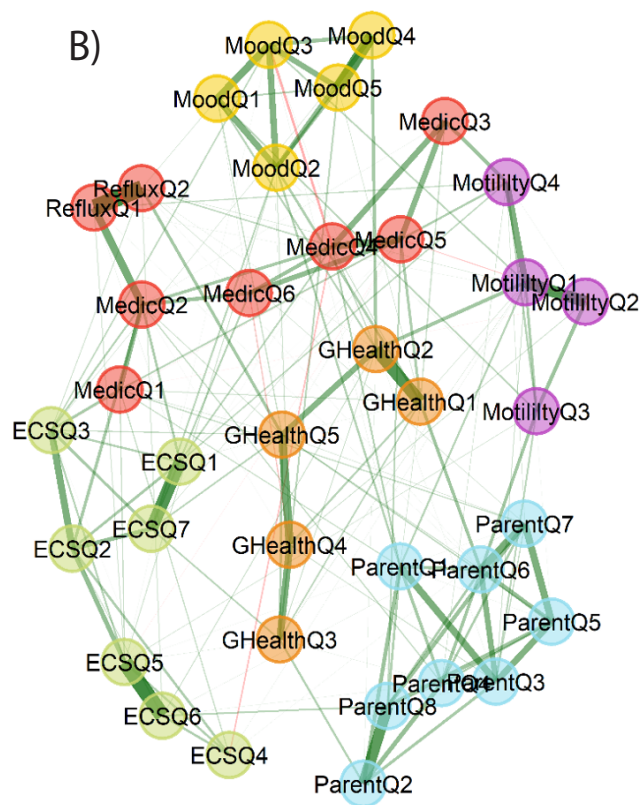

Supplement: Supplementary file 2 — Additional file 2: Figure S2. Exploratory Graph Analysis of the GHQ. A. Final GHS model based on Confirmatory Factor Analysis. B. Proposed Exploratory Graph Analysis model. ECS: Eating-Chewing-Swallowing, GHealth: General Health, Medic: Medication, Q: Question, Parent: Parenting. [file 13023_2024_3022_MOESM2_ESM.pdf]
