# Supplementary material for: Development and validation of parent-reported gastrointestinal health scale in MECP2 duplication syndrome
Source: Orphanet J Rare Dis. 2024 Feb 9;19:52. doi: 10.1186/s13023-024-03022-2 (PMC10854118; doi:10.1186/s13023-024-03022-2)
Supplement: Supplementary file 3 — Additional file 3. Details of the item reduction/retention studies based on CFA, parent-reports and expert opinion. [file 13023_2024_3022_MOESM3_ESM.pdf]

Item	Relevance 1<25%	Importance > 60 %	REVISED Relevance 1<33%
I.a. General Health/Pain Q1	20.8	69.8	20.8
I.a. General Health/Pain Q2	22.6	69.8	22.6
I.a. General Health/Pain Q3	55.7	47.1	55.7
I.a. General Health/Pain Q4	60.4	35.8	60.4
I.a. General Health/Pain Q5	52.8	39.6	52.8
II.a. Eating , Chewing, and Swallowing Q1	36.8	56.6	36.8
II.a. Eating , Chewing, and Swallowing Q2	17	74.5	17
II.a. Eating , Chewing, and Swallowing Q3	23.6	63.2	23.6
II.a. Eating , Chewing, and Swallowing Q4	12.3	70.8	12.3
II.a. Eating , Chewing, and Swallowing Q5	17	72.6	17
II.a. Eating , Chewing, and Swallowing Q6	18.9	71.7	18.9
II.a. Eating , Chewing, and Swallowing Q7	51.9	45.3	51.9
II.a. Eating , Chewing, and Swallowing Q8	28.3	57.6	28.3
II.a. Eating , Chewing, and Swallowing Q9	45.3	47.2	45.3
III.a. Reflux Q1	34	60.4	34
III.a. Reflux Q2	46.2	47.2	46.2
III.a. Reflux Q3	32.1	53.8	32.1
IV.a. Gas and Bloating Q1	31.1	49.1	31.1
IV.a. Gas and Bloating Q2	18.9	62.2	18.9
IV.a. Gas and Bloating Q3	20.8	61.4	20.8
IV.a. Gas and Bloating Q4	25.5	44.4	25.5
IV.a. Gas and Bloating Q5	21.7	53.8	21.7
V.a. Diarrhea and Constipation Q1	35.8	50.9	35.8
V.a. Diarrhea and Constipation Q2	32.1	52.8	32.1
V.a. Diarrhea and Constipation Q3	5.7	87.8	5.7
V.a. Diarrhea and Constipation Q4	11.3	81.1	11.3
V.a. Diarrhea and Constipation Q5	5.7	77.3	5.7
V.a. Diarrhea and Constipation Q6	12.3	85.9	12.3
VI.a. Personality and Mood Q1	33	51	33
VI.a. Personality and Mood Q2	34.9	50	34.9
VI.a. Personality and Mood Q3	32.1	46.6	32.1
VI.a. Personality and Mood Q4	30.2	55.7	30.2
VI.a. Personality and Mood Q5	18.9	62.2	18.9
VII.a. Medications Q1	60.4	45.3	60.4
VII.a. Medications Q2	50.9	48.1	50.9
VII.a. Medications Q3	55.7	42.4	55.7
VII.a. Medications Q4	68.9	34.9	68.9
VII.a. Medications Q5	59.4	36.8	59.4
VII.a. Medications Q6	65.1	35.8	65.1
VII.a. Medications Q7	40.6	55.7	40.6
VII.a. Medications Q8	28.3	68.9	28.3
VII.a. Medications Q9	68.9	30.2	68.9
VIII.a. Surgery Q1	58.5	51	58.5
VIII.a. Surgery Q2	70.8	36.8	70.8
VIII.a. Surgery Q3	82.1	29.2	82.1

VIII.a. Surgery Q4	77.4	32	77.4
VIII.a. Surgery Q5	80.2	29.2	80.2
IX.a. Parenting Q1	16	58.5	16
IX.a. Parenting Q2	8.5	76.4	8.5
IX.a. Parenting Q3	23.6	57.5	23.6
IX.a. Parenting Q4	13.2	71.7	13.2
IX.a. Parenting Q5	29.2	58.5	29.2
IX.a. Parenting Q6	25.5	51.99	25.5
IX.a. Parenting Q7	23.6	53.7	23.6
IX.a. Parenting Q8	13.2	68.9	13.2
Total Included	**16**	**16**	**30**
			`

REVISED Importance > 47 %	Mean Score>1.5 Relevance	Mean Score>1.5 Importance	Parent based selection
69.8	2.54	3.02	GHealthQ1
69.8	2.54	3.00	GHP_a2
47.1	1.92	2.37	GHP_a3
35.8	1.68	2.16	GHP_a4
39.6	1.82	2.23	GHP_a5
56.6	2.44	2.66	ECS_a1
74.5	3.11	3.16	ECS_a2
63.2	2.76	2.88	ECS_a3
70.8	3.09	3.08	ECS_a4
72.6	2.97	3.11	ECS_a5
71.7	2.93	3.11	ECS_a6
45.3	2.25	2.35	ECS_a7
57.6	2.73	2.75	ECS_a8
47.2	2.23	2.36	ECS_a9
60.4	2.43	2.78	REFL_a1
47.2	2.04	2.47	REFL_a2
53.8	2.36	2.61	REFL_a3
49.1	2.38	2.48	GB_a1
62.2	2.67	2.82	GB_a2
61.4	2.67	2.86	GB_a3
44.4	2.42	2.45	GB_a4
53.8	2.65	2.69	GB_a5
50.9	2.42	2.56	DC_a1
52.8	2.44	2.61	DC_a2
87.8	3.45	3.54	DC_a3
81.1	3.19	3.29	DC_a4
77.3	3.23	3.25	DC_a5
85.9	3.38	3.49	DC_a6
51	2.30	2.54	PM_a1
50	2.21	2.48	PM_a2
46.6	2.25	2.45	PM_a3
55.7	2.28	2.63	PM_a4
62.2	2.60	2.84	PM_a5
45.3	1.91	2.34	Med_a1
48.1	2.23	2.46	Med_a2
42.4	2.00	2.28	Med_a3
34.9	1.71	2.07	Med_a4
36.8	1.87	2.15	Med_a5
35.8	1.80	2.09	Med_a6
55.7	2.45	2.65	Med_a7
68.9	2.87	3.01	Med_a8
30.2	1.72	1.92	Med_a9
51	2.09	2.57	SRG_a1
36.8	1.75	2.14	SRG_a2
29.2	1.46	1.93	SRG_a3

32	1.59	2.02	SRG_a4
29.2	1.46	1.93	SRG_a5
58.5	2.77	2.83	PAR_a1
76.4	3.23	3.22	PAR_a2
57.5	2.60	2.74	PAR_a3
71.7	3.02	3.08	PAR_a4
58.5	2.58	2.75	PAR_a5
51.99	2.56	2.59	PAR_a6
53.7	2.63	2.70	PAR_a7
68.9	3.05	3.07	PAR_a8
**30**			**30**

Factor Loadings	Expert Opinion Inclusion/Exclusion	FINAL
GHP_a1	Included	Included
GHP_a2	Included	Included
GHP_a3	Included	Included
GHP_a4	Excluded	Excluded
GHP_a5	Excluded	Excluded
ECS_a1	Included	Included
ECS_a2	Included	Included
ECS_a3	Included	Included
ECS_a4	Included	Included
ECS_a5	Included	Included
ECS_a6	Included	Included
ECS_a7	Included	Included
ECS_a8	Included	Included
ECS_a9	Excluded	Excluded
REFL_a1	Included	Included
REFL_a2	Included	Included
REFL_a3	Included	Included
GB_a1	Excluded	Excluded
GB_a2	Excluded	Excluded
GB_a3	Excluded	Excluded
GB_a4	Excluded	Excluded
GB_a5	Excluded	Excluded
DC_a1	Excluded	Excluded
DC_a2	Excluded	Excluded
DC_a3	Included	Included
DC_a4	Included	Included
DC_a5	Included	Included
DC_a6	Included	Included
PM_a1	Included	Included
PM_a2	Included	Included
PM_a3	Included	Included
PM_a4	Included	Included
PM_a5	Included	Included
Med_a1	Included	Included
Med_a2	Included	Included
Med_a3	Included	Included
Med_a4	Included	Included
Med_a5	Excluded	Excluded
Med_a6	Included	Included
Med_a7	Included	Included
Med_a8	Included	Included
Med_a9	Excluded	Excluded
SRG_a1	Excluded	Excluded
SRG_a2	Excluded	Excluded
SRG_a3	Excluded	Excluded

SRG_a4	Excluded	Excluded
SRG_a5	Excluded	Excluded
PAR_a1	Included	Included
PAR_a2	Included	Included
PAR_a3	Included	Included
PAR_a4	Included	Included
PAR_a5	Included	Included
PAR_a6	Included	Included
PAR_a7	Included	Included
PAR_a8	Included	Included
**44**	**38**	
