# Supplementary material for: Development and validation of parent-reported gastrointestinal health scale in MECP2 duplication syndrome
Source: Orphanet J Rare Dis. 2024 Feb 9;19:52. doi: 10.1186/s13023-024-03022-2 (PMC10854118; doi:10.1186/s13023-024-03022-2)
Supplement: Supplementary file 4 — Additional file 4. Collinearity values for factors and each item of Gastrointestinal Health Scale. [file 13023_2024_3022_MOESM4_ESM.docx]

| **Factor Based** | | | | | | | |
| --- | --- | --- | --- | --- | --- | --- | --- |
| **Parent** | | | | | |  |  |
| General health | Eating-Chewing-Swallowing | Reflux | Motility | Mood | Medication |  |  |
| 2.159 | 1.542 | 1.827 | 1.324 | 1.756 | 1.446 |  |  |
|  |  |  |  |  |  |  |  |
| **Item based** | | | | | | | |
| **General Health** | | |  |  |  |  |  |
| General Health 1 | General Health 2 | General Health 3 |  |  |  |  |  |
| 2.674 | 2.707 | 1.324 |  |  |  |  |  |
| **Eating-Chewing-Swallowing (ECS)** | | | | | | | |
| ECS 1 | ECS 2 | ECS 3 | ECS 4 | ECS 5 | ECS 6 | ECS 7 | ECS 8 |
| 2.124 | 2.052 | 1.851 | 1.203 | 2.689 | 2.403 | 2.154 | 1.536 |
| **Reflux** | | |  |  |  |  |  |
| Reflux 1 | Reflux 2 | Reflux 3 |  |  |  |  |  |
| 2.331 | 2.516 | 1.372 |  |  |  |  |  |
| **Motility** | | | |  |  |  |  |
| Motility 1 | Motility 2 | Motility 3 | Motility 4 |  |  |  |  |
| 2.034 | 1.695 | 1.352 | 1.376 |  |  |  |  |
| **Mood** | | | | |  |  |  |
| Mood 1 | Mood 2 | Mood 3 | Mood 4 | Mood 5 |  |  |  |
| 2.181 | 2.867 | 2.713 | 2.76 | 2.766 |  |  |  |
| **Medication** | | | | | | |  |
| Medication 1 | Medication 2 | Medication 3 | Medication 4 | Medication 5 | Medication 6 | Medication 7 |  |
| 1.177 | 1.396 | 1.176 | 1.286 | 1.332 | 1.224 | 1.126 |  |
| **Parenting** | | | | | | | |
| Parenting 1 | Parenting 2 | Parenting 3 | Parenting 4 | Parenting 5 | Parenting 6 | Parenting 7 | Parenting 8 |
| 2.998 | 3.845 | 4.275 | 2.158 | 3.549 | 3.925 | 3.706 | 4.17 |

**Table S2: Collinearity values for factors and each item of Gastrointestinal Health Scale**

ECS: Eating-Chewing-Swallowing
